# Camera trap survey of mammal diversity and activity rhythms of threatened species in a subtropical forest of Huangshan Mountain, China

**DOI:** 10.3897/BDJ.14.e184017

**Published:** 2026-02-19

**Authors:** Wei Zhao, Meng-Meng Li, Xin-Rui Yan, Zheng Zhang, Xiao-Chun Chen, Zhao-Qian Chang, Jia-Long Chen, Jun-Jie Ma, Zhe-Ping Xu, Jin-Min Chen, Ping Shin Lee, Fang Zhang

**Affiliations:** 1 The Anhui Provincial Key Laboratory of Biodiversity Conservation and Ecological Security in the Yangtze River Basin, College of Life Sciences, Anhui Normal University, Wuhu 241000, Anhui, China The Anhui Provincial Key Laboratory of Biodiversity Conservation and Ecological Security in the Yangtze River Basin, College of Life Sciences, Anhui Normal University Wuhu 241000, Anhui China; 2 Shenzhen Taohuayuan Conservation Foundation, Shenzhen 518000, Guangdong, China Shenzhen Taohuayuan Conservation Foundation Shenzhen 518000, Guangdong China; 3 Huangshan Jiulongfeng Nature Reserve Management Station, Huangshan 245000, Anhui, China Huangshan Jiulongfeng Nature Reserve Management Station Huangshan 245000, Anhui China; 4 Department of Information Resources Management, School of Economics and Management, University of Chinese Academy of Sciences, Beijing 100190, China Department of Information Resources Management, School of Economics and Management, University of Chinese Academy of Sciences Beijing 100190 China; 5 National Science Library, Chinese Academy of Sciences, Beijing 100190, China National Science Library, Chinese Academy of Sciences Beijing 100190 China

**Keywords:** community composition, conservation, seasonal altitudinal migration, species of conservation concern

## Abstract

**Background:**

Understanding the activity rhythms of threatened mammals is crucial for designing effective conservation strategies. However, the systematic studies on mammals in Huangshan Mountain, one of China’s most biodiverse regions, are limited. Therefore, the current study aimed to document the diversity of medium- and large-sized mammals alongside the daily and seasonal activity rhythms of threatened species in the Jiulongfeng Nature Reserve of Huangshan Mountain.

**New information:**

A survey employing 32 infrared cameras over 7964 camera-days from March 2022 to March 2023 yielded 7625 independent detections of 15 species belonging to five orders, 13 genera and 10 families. The top five species with the highest relative abundance index (RAI) values were Reeves’s Muntjac (*Muntiacus reevesi*), Wild Boar (*Sus scrofa*), Tibetan Macaque (*Macaca thibetana huangshanensis*), Masked Palm Civet (*Paguma larvata*), and Malayan Porcupine (*Hystrix brachyura*). We describe the diel activity rhythms and seasonal altitudinal migration patterns of seven focal threatened species. The daily activity patterns of Reeves’s Muntjacs, Black Muntjacs (*Muntiacus crinifrons*) and Tibetan Macaques were bimodal and predominantly diurnal, whereas Masked Palm Civets, Malayan Porcupines, Hog Badgers (*Arctonyx collaris*), and Serows (*Capricornis sumatraensis*) were nocturnal. Activity peaks also shifted seasonally. We further observed seasonal altitudinal migration patterns: diurnal mammals, such as Reeves's Muntjacs, Black Muntjacs and Tibetan Macaques, frequented mid-altitude in summer, while nocturnal species, such as Hog Badgers, Malayan Porcupines and Masked Palm Civets were more common at mid-altitude in winter. This pattern could be attributed to thermoregulation and other ecological factors. This work contributes valuable baseline data on mammalian biodiversity and behavioural ecology in an understudied region, with implications for the conservation management of protected areas in subtropical China.

## Introduction

As keystone components of biodiversity, mammals play a vital role in maintaining the balance and stability of ecosystems (Stevens et al. 2019). However, with the continuous expansion of human activities and the deterioration of the environment, many mammal species are threatened at present and effective monitoring is essential for the conservation of local animal diversity (Ceballos and Ehrlich 2006). In assessments of mammalian diversity, activity rhythms are important parameters, as they can reveal the habits and behavioural patterns of mammals (Amstrup and Beecham 1976). The variation in activity rhythms of animals across times and seasons can reflect the adaptations, circadian rhythms, seasonal distribution patterns and other characteristics of individual animals (Azevedo et al. 2018).

In recent years, the infrared camera monitoring method has been widely used in mammal diversity surveys and activity rhythm studies in protected areas owing to its attributes of continuous monitoring, low interference and non-invasiveness (Deng et al. 2021, Zhou et al. 2022, Pang et al. 2025, Yang et al. 2025). Infrared camera surveys are especially convenient for monitoring the density and distribution of threatened species and revealing their ecological habits and behavioural patterns (Burton et al. 2015, Ikeda et al. 2016, Ogurtsov et al. 2018). For example, this method has successfully revealed the activity rhythms of threatened mammals, such as the Black Muntjac *Muntiacus crinifrons* (P.L. Sclater, 1885) (Zhang et al. 2012), Reeves’s Muntjac *Muntiacus reevesi* (W. Ogilby, 1839) (Kong et al. 2024), Serow *Capricornis sumatraensis* (Bechstein, 1799) (Jia et al. 2022), Masked Palm Civet *Paguma larvata* (Griffith, 1822) (Duan et al. 2024), Hog Badger *Arctonyx collaris* F. Cuvier in É. Geoffroy Saint-Hilaire & F. Cuvier, 1825 (Jiang et al. 2021), Malayan Porcupine *Hystrix brachyura* Linnaeus, 1758 (Dai et al. 2021) and Tibetan Macaque *Macaca thibetana huangshanensis* Jiang Xuelong & Wang Qishan in Jiang Xuelong, Wang Yingxiang and Wang Qishan, 1996 (Jia et al. 2022).

Despite these advancements, systematic research on mammal activity rhythms in many highly biodiverse regions, including the Huangshan Mountain, remains scarce. Recognised by the IUCN as one of the world's 108 biodiversity centres and a national conservation priority in China, the Huangshan Mountain supports a rich range of mammalian fauna (Huang et al. 2015). However, foundational data on the activity patterns of its threatened mammal community are lacking, hindering the development of management plans for its protected areas. The Jiulongfeng Nature Reserve (JLF), located within the Huangshan Mountain range, is characterised by its high mountains, dense subtropical forests and deep gorges, all of which provide critical habitats for a variety of wildlife (Wu 2019). To date, comprehensive research on the activity rhythms of mammals in the Jiulongfeng Reserve has not been conducted.

## General description

### Purpose

The study aimed to document the community composition of medium- to large-sized mammals and investigated the activity rhythms of threatened species in the JLF using camera-trapping data, thereby providing baseline data on the unique wild mammal species of the study area.

## Project description

### Study area description

The JLF is located between 117°56'32"~118°03'55"E longitude and 30°04'05"~30°10'23"N latitude. The annual average temperature is 15.5°C; the extreme maximum temperature is 40.7°C and the extreme minimum temperature is −14.7°C. The annual average precipitation is about 1759 mm; the total area is 2720 km^2^ and the highest altitude is 1281 m. The territory is characterised by continuous mountains, overlapping peaks, a mild climate and abundant rainfall (Wu 2019).

### Design description

**Figure 1.**
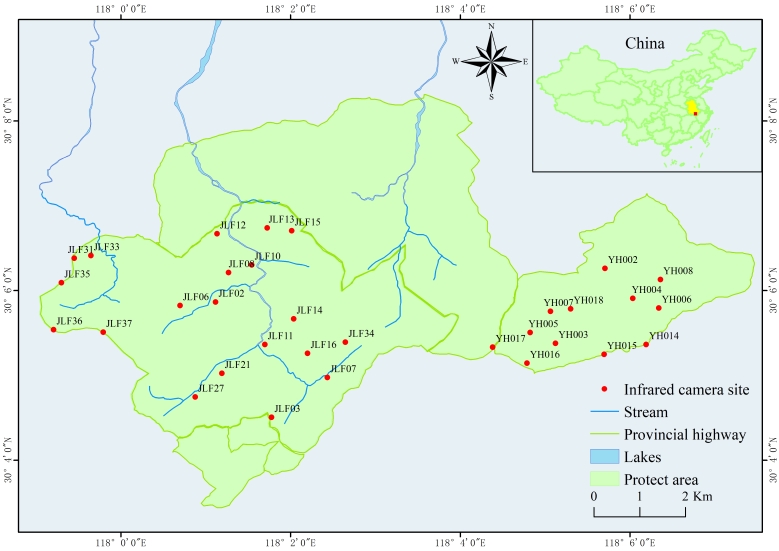
Location of camera traps in the Jiulongfeng Nature Reserve.

We employed 32 infrared camera traps (Yianws L710, Shenzhen, China) to conduct wild mammal surveys from March 2022 to March 2023 (Fig. 1). Based on the observed signs of animal activities (e.g. trails, scat, nests and feeding traces), forest-land locations that were more open and closer to water sources were selected to install the infrared cameras and information, such as the camera shooting time, ambient temperature, deployment habitat and altitude of each camera site, was recorded.

### Funding

This research was funded by the National Natural Science Foundation of China (NSFC 32470458; 32001222), Anhui Provincial Key Laboratory of the Conservation and Exploitation of Biological Resources (No. 591601) and the Innovation and Entrepreneurship Training Programme for Undergraduates Students (S202410370265; 202510370712).

## Sampling methods

### Study extent

Species were identified by analysing the captured photos and videos. We placed each camera on relatively flat terrain 50 cm above the ground and removed all vegetation and debris from the camera view. The spacing between the cameras was at least 200 m to ensure spatial independence. All cameras were monitored 24 hours a day and all camera parameters were set uniformly, including the date and shooting mode (3 photos + 1 video mode).

### Quality control

To ensure accurate species identification, the authors thoroughly reviewed the photos and cross-checked the nomenclature, consulting the IUCN Red List (IUCN 2025) and considering each species' conservation status. Afterwards, all ambiguous records that could not be identified at the genus level were excluded from the final analysis. The records belonging to Muridae were thus removed from the dataset, as we were unable to identify them at the species level.

### Step description

The photo and video data captured by the infrared cameras were archived and saved according to the camera numbers and corresponding folders were created. Information, such as the file numbers, formats and shooting dates and times, was extracted from each folder and recorded in Excel. Species identification was conducted using the image data and independent detections were screened. An independent photo refers to adjacent images of the same individual at the same camera site with an interval of at least 30 minutes (O'Brien et al. 2006).

The richness of each wild animal species was represented by RAI (Relative Abundance Index, i.e. the number of independent detections per 100 camera days) and multiple α-diversity indices were used to evaluate the differences in species richness between low-altitude and mid-altitude areas (Shannon 1948, Simpson 1949). The dilution curves were constructed using the Chao 1 method implemented in EstimateS 9.1.0 (Colwell 2019) and the sampling saturation of the infrared camera monitoring was explored. To estimate the daily and seasonal activity patterns of wild animals, we used kernel density analysis (Ridout and Linkie 2009) via the ‘activity’ package for R (R Core Team 2025). According to the climatic characteristics of the study area, 18:00–06:00 h was taken as the night-time and the night-time relative abundance index (NRAI) of each species was calculated. If the NRAI was greater than 13/24 (0.542), the species was considered a nocturnal animal (Li et al. 2014).

The altitude of the survey area in this investigation ranged from 433 m to 1203 m. Due to the limited altitude range of the camera sites set in this study, the altitude was divided into low altitude (400–780 m) and mid-altitude (780–1210 m). The four seasons were divided as follows: spring from March to May, summer from June to August, autumn from September to November and winter from December to February of the following year.

## Geographic coverage

### Description

The Jiulongfeng Nature Reserve of Huangshan Mountain, Anhui, China.

### Coordinates

 and 30.06806 and 30.17306 Latitude Latitude; and 117.94222 and 118.06528 Longitude Longitude.

## Taxonomic coverage

### Description

In this study, we covered the following taxonomic groups: Class: Mammalia; Orders: Lagomorpha, Carnivora, Rodentia, Artiodactyla and Primates; Families: Leporidae, Mustelidae, Viverridae, Herpestidae, Suidae, Cervidae, Bovidae, Cercopithecidae and Sciuridae.

### Taxa included

**Table:** 

Rank	Scientific Name	Common Name
class	Mammalia	Mammals
order	Lagomorpha	Hares
order	Carnivora	Carnivores
order	Artiodactyla	Ruminants
order	Primates	Primates
order	Rodentia	Rodents
family	Leporidae	Hares
family	Mustelidae	Mustelids
family	Viverridae	Civets
family	Herpestidae	Mongooses
family	Suidae	Pigs
family	Cervidae	Deers
family	Bovidae	Bovids
family	Cercopithecidae	Monkeys
family	Sciuridae	Squirrels
family	Hystricidae	Porcupines

## Temporal coverage

**Data range:** 2022-3-24 – 2023-3-31.

## Usage licence

### Usage licence

Other

### IP rights notes

Creative Commons Attribution 4.0 International (CC-BY-4.0).

## Data resources

### Data package title

Camera trap survey of mammal diversity in a subtropical forest of Huangshan Mountain, China.

### Resource link

https://doi.org/10.15468/94t4wd

### Alternative identifiers

https://www.gbifchina.org.cn/resource?r=zw20000919

### Number of data sets

1

### Data set 1.

#### Data set name

Camera trap survey of mammal diversity in a subtropical forest of Huangshan Mountain, China.

#### Data format

Darwin Core Archive

#### Download URL

https://www.gbifchina.org.cn/archive.do?r=zw20000919

#### Data format version

version 1.6

#### Description

The dataset was published in GBIF (Zhao and Chen 2025). The following data table includes all the records for which a taxonomic identification of the species was possible. The dataset submitted to GBIF is structured as a sample event dataset, with two tables: event and occurrences. The data in this sampling event resource have been published as a Darwin Core Archive (DwC-A), which is a standardised format for sharing biodiversity data as a set of one or more data tables. The core data table contains 32 records (eventID). One extension data table also exists with 7625 occurrences.

**Data set 1.** 

Column label	Column description
id (Event Core, Occurrence Extension)	Unique identifier.
eventID (Event Core, Occurrence Extension)	A unique identifier linking events and occurrences.
samplingProtocol (Event Core)	The sampling method used.
samplingEffort (Event Core)	The number of camera-trap days expended during an event.
eventDate (Event Core, Occurrence Extension)	Date or date range the record was collected.
locationID (Event Core)	An identifier of the camera location.
country (Event Core)	Country in which camera location occurs.
countryCode (Event Core)	ISO code of the country.
stateProvince (Event Core)	Name of the region in which camera location occurs.
decimalLatitude (Event Core)	The geographic latitude, in decimal degrees.
decimalLongitude (Event Core)	The geographic longitude, in decimal degrees.
geodeticDatum (Event Core)	The reference point for the various coordinate systems used in mapping the earth.
coordinateUncertaintyInMetres (Event Core)	Uncertainty of the coordinates, in metres.
occurrenceID (Occurrence Extension)	Unique identifier of the record.
basisOfRecord (Occurrence Extension)	The specific nature of the data record.
occurrenceStatus (Occurrence Extension)	A statement about the presence or absence of a taxon at a location.
kingdom (Occurrence Extension)	Kingdom name in which the taxon is classified.
phylum (Occurrence Extension)	Phylum name in which the taxon is classified.
class (Occurrence Extension)	Class name in which the taxon is classified.
order (Occurrence Extension)	Order name in which the taxon is classified.
family (Occurrence Extension)	Family name in which the taxon is classified.
scientificName (Occurrence Extension)	The full scientific name includes author and year.
infraspecificEpithet (Occurrence Extension)	Subspecies name in which the taxon is classified.
taxonRank (Occurrence Extension)	The taxonomic rank of the most specific name in the scientificName.
dynamicProperties (Occurrence Extension)	IUCN Red List status of the taxon.
vernacularName (Occurrence Extension)	The common name of a species.

## Additional information

### Results

**Figure 2.**
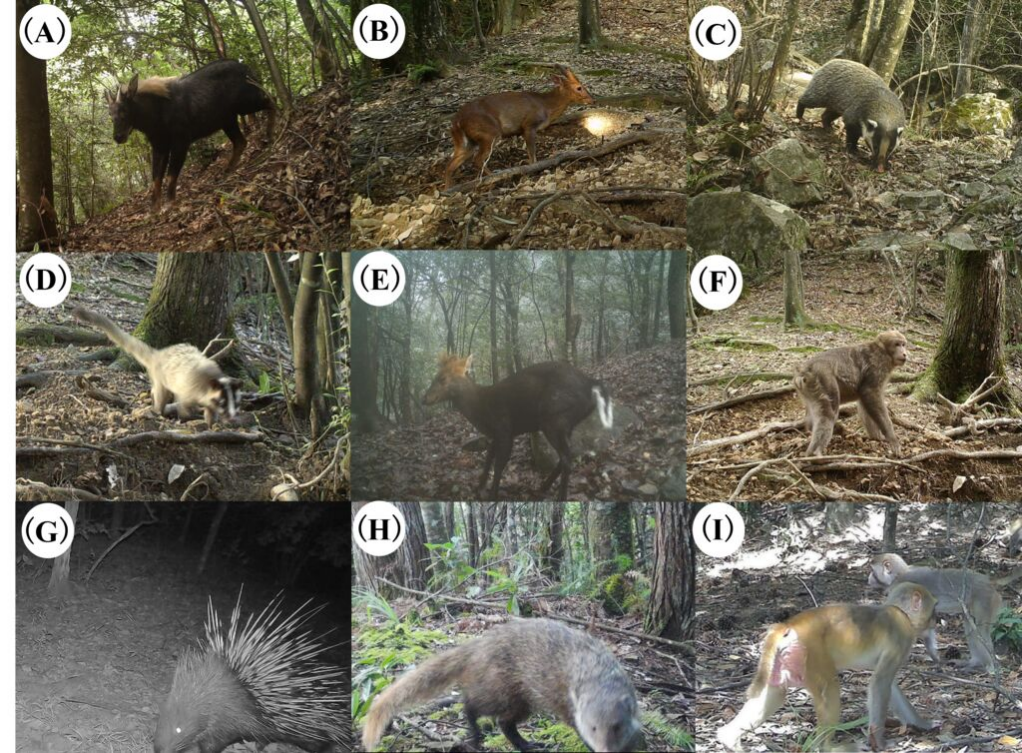
Mammalian species captured by infrared cameras at the Jiulongfeng Nature Reserve: **A** Serow (*Capricornis sumatraensis*); **B** Reeves’s Muntjac (*Muntiacus reevesi*); **C** Hog Badger (*Arctonyx collaris*); **D** Masked Palm Civet (*Paguma larvata*); **E** Black Muntjac (*Muntiacus crinifrons*); **F** Tibetan Macaque (*Macaca thibetana huangshanensis*); **G** Malayan Porcupine (*Hystrix brachyura*); **H** Crab-eating Mongoose (*Urva urva*); **I** Rhesus Macaque (*Macaca mulatta*).

The survey was a total effective working period of 7964 days, producing a total of 7625 independent mammal photo detections. Accordingly, 15 mammal species were identified (Fig. 2), with the species belonging to five orders and 10 families, including six carnivores, four even-toed ungulates, two primates, two rodents and one rabbit, accounting for 28.85% of the total number of mammal species recorded from JLF (Suppl. material 1). Amongst these, there was one species of Black Muntjac, a national Class I protected wild animal and four species of wild animals under national Class II protection: the Yellow-throated Marten *Martes flavigula* (Boddaert, 1785), the Serow, the Rhesus Macaque *Macaca mulatta* (E.A.W. von Zimmermann, 1780) and the Tibetan Macaque. Three species were listed as Vulnerable (VU) on the IUCN: the Black Muntjac, the Serow and the Hog Badger. The one species listed as Near Threatened (NT) was the Tibetan Macaque. The top five species with high RAI were Reeves’s Muntjac (RAI = 43.70), the Wild Boar *Sus scrofa* Linnaeus, 1758 (RAI = 17.83), the Tibetan Macaque (RAI = 10.01), the Masked Palm Civet (RAI = 6.47) and the Malayan Porcupine (RAI = 6.24) (Table 1).

**Figure 3.**
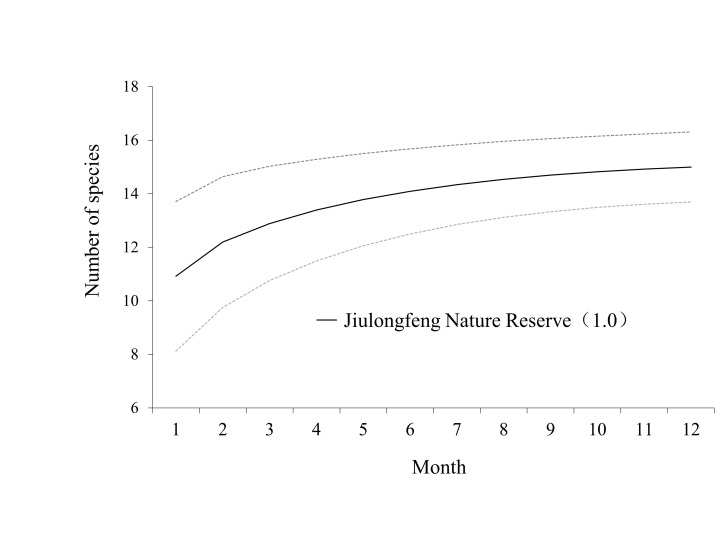
Expected species richness curve and sampling completeness ratios (in parentheses) at the Jiulongfeng Nature Reserve, with the dotted lines representing the 95% confidence intervals of the expected species richness in the Jiulongfeng Nature Reserve.

The number of species monitored by infrared cameras was positively correlated with the monitoring time. The rarefaction curve achieved full saturation (completeness = 1.0) (Fig. 3). After all cameras were placed in the wild for one month, a total of 12 species of mammals were captured. After five months, 14 species of mammals were photographed. The number of species captured increased with an increase in monitoring time, but the trend of the increase in the number of species slowed down after three months.

We compared mammal α-diversity between two altitudinal zones, with 16 camera sites in each quadrat and the results showed that both the diversity and evenness indices were comparatively higher in the mid-altitude zone (Table 2).

**Figure 4.**
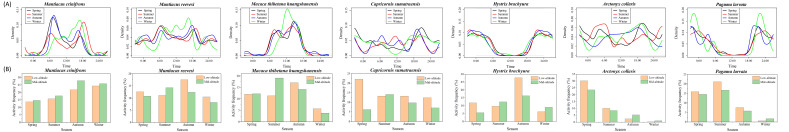
Daily activity patterns (A) and seasonal altitudinal migration (B) of seven threatened species in the Jiulongfeng Nature Reserve.

The overall daily activity patterns of Reeves’s Muntjacs, Black Muntjacs and Tibetan Macaques were similar and the activity curve was "M"-shaped, demonstrating a typical bimodal pattern, with clear activity peaks in the morning and dusk (Fig. 4A). Black Muntjacs showed similar diurnal activity patterns in spring, summer and autumn, but became more crepuscular in winter, with a single peak at 18:00 h. While Reeves’s Muntjacs and Tibetan Macaques shared similar activity frequencies in spring, summer and autumn, their winter patterns diverged: Tibetan Macaques displayed a single peak, whereas Reeves’s Muntjacs showed three peaks, including a minor one at noon (12:00 h). Masked Palm Civets and Malayan Porcupines were both nocturnal, with a "U"-shaped activity curve and a pronounced daytime lull. However, the seasonal consistency of their peak activity differed: Malayan Porcupines maintained similar peaks across all seasons, while Masked Palm Civets peaks varied, though they predominantly occurred between 22:00 h and 03:00 h. Serows and Hog Badgers both exhibited fluctuating activity rhythms, with primary peaks occurring between 04:00–06:00 h and 18:00–20:00 h.

**Figure 5.**
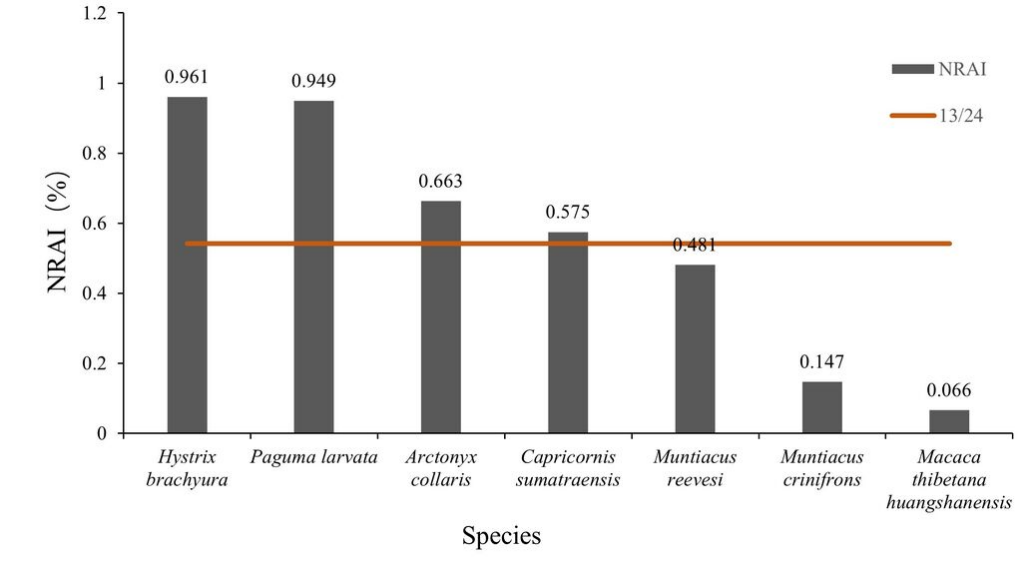
Nocturnal indices of seven threatened species in the Jiulongfeng Nature Reserve.

The nocturnal relative abundance index (NRAI) for the seven focal species was calculated as 0.961, 0.949, 0.663, 0.575, 0.481, 0.147 and 0.066 (Fig. 5). Based on a threshold of 13/24, Reeves’s Muntjacs, Black Muntjacs and Tibetan Macaques were classified as diurnal, whereas Malayan Porcupines, Masked Palm Civets and Hog Badgers were nocturnal. The Serow, with an NRAI of 0.575, was not strictly nocturnal.

**Figure 6.**
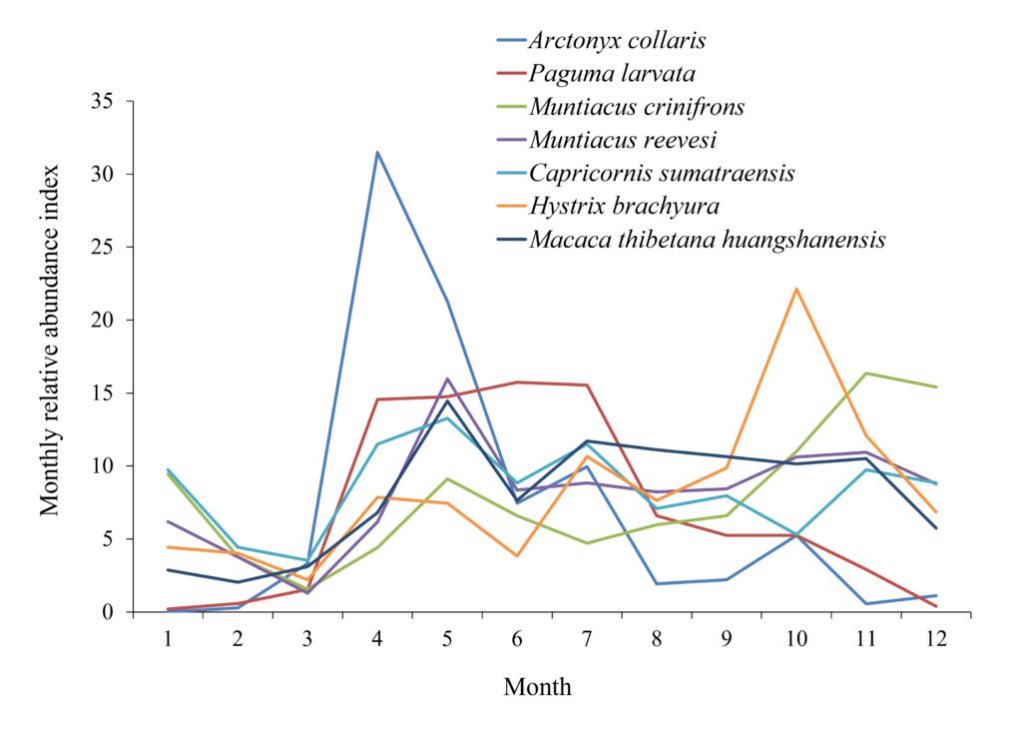
Annual activity patterns of seven threatened species in the Jiulongfeng Nature Reserve.

Monthly relative activity intensity differed significantly for Hog Badgers and Tibetan Macaques, but not for Masked Palm Civets, Black Muntjacs, Reeves’s Muntjacs, Serows or Malayan Porcupines (Suppl. material 2). Notably, Hog Badgers were only active in spring and summer; the activity frequency gradually increased from March, peaked in April and subsequently declined. Activity peaks for other species were observed in Tibetan Macaques and Reeves’s Muntjacs (May), Malayan Porcupines (October), Black Muntjacs (November) and Serows (summer). Masked Palm Civets had the highest activity intensity in spring, which decreased markedly after July (Fig. 6).

Significant seasonal variation in altitude selection was observed for Hog Badgers, Masked Palm Civets and Black Muntjacs, but not for Reeves’s Muntjacs, Serows, Malayan Porcupines and Tibetan Macaques across seasons (Suppl. material 2). Hog Badgers tended to inhabit low altitudes in spring and summer and mid-altitudes in autumn and winter, opposite to the altitude distribution of Tibetan Macaques. Masked Palm Civets chose low altitude in spring, summer and autumn and mid-altitude in winter. Reeves’s Muntjacs and Serows tended to inhabit mid-altitude in summer and low altitude in spring, autumn and winter. Malayan Porcupines chose low altitudes in spring and autumn and mid-altitudes in summer and winter. Black Muntjacs tended to be at mid-altitude throughout the year (Fig. 4B).

### Discussion

Our dataset establishes a crucial baseline for future ecological research, long-term monitoring and informed conservation management in the Huangshan Mountain. The capture of 15 medium- to large-sized species underscores the considerable conservation value in JLF and the effectiveness of our survey. The rarefaction curve confirms that our sampling effort was sufficiently robust to adequately characterise the core mammal community at the camera sites. The dominance of Wild Boars and Reeves's Muntjacs in the RAI rankings is consistent with findings from other subtropical Chinese Reserves (Burton et al. 2015, Liu et al. 2017, Zhou et al. 2018, Zhu 2022, Xu et al. 2023).

The analysis of activity rhythms revealed profound interspecific and seasonal variations, reflecting distinct adaptive strategies. For example, the strict nocturnality of Malayan Porcupines and Masked Palm Civets aligns with their foraging ecology and this may represent a behavioural adaptation to avoid diurnal human activity (Yuan and Kong 2011). In contrast, the bimodal patterns of Reeves's Muntjacs and Tibetan Macaques, which shifted to include heightened mid-day activity in winter, are an energy-saving strategy to capitalise on warmer daytime temperatures and compensate for the reduced foraging time during shorter days (Chen et al. 2022).

The observed seasonal altitudinal migrations suggest a complex trade-off between multiple ecological factors. The Hog Badgers and Masked Palm Civets descending to lower altitudes in spring and summer could be driven by the greater abundance of food resources (Wang et al. 2009). The year-round preference of Black Muntjacs for mid-altitude indicates specific habitat requirements that may be related to denser understorey cover that provides both forage and refuge from disturbance (Moen 1976, Bao et al. 2006). Future research should integrate fine-scale vegetation mapping, the direct quantification of human disturbance and climate data towards a mechanistic understanding of these ecological dynamics.

## Supplementary Material

37440F5D-0579-59AC-BCD8-2AAE9297B6BC 10.3897/BDJ.14.e184017.suppl1 Supplementary material 1 List of mammal species Data type Table Brief description List of mammal species recorded at the Jiulongfeng Nature Reserve, Huangshan of Anhui Province. File: oo_1513434.pdf https://binary.pensoft.net/file/1513434 Wei Zhao

9826AD15-3962-5A98-A7A6-57A812D8B675 10.3897/BDJ.14.e184017.suppl2 Supplementary material 2 Differences in the number of detections Data type Table Brief description Differences in the number of detections across months and differences in altitude selection across seasons for the seven threatened species. File: oo_1473445.xlsx https://binary.pensoft.net/file/1473445 Wei Zhao

## Figures and Tables

**Table 1.** Species diversity of mammals in the Jiulongfeng Nature Reserve.

Species	Common name	IUCN Red List	National Key Protection Level	Number of independent photos	Relative abundance index
** LAGOMORPHA **					
** Leporidae **					
*Lepus sinensis* J.E. Gray, 1832	Chinese Hare	LC	-	24	0.30
** CARNIVORA **					
** Mustelidae **					
*Martes flavigula* (Boddaert, 1785)	Yellow-throated Marten	LC	II	49	0.62
*Mustela sibirica* Pallas, 1773	Siberian Weasel	LC	-	4	0.05
*Meles meles* (Linnaeus, 1758)	European Badger	LC	-	6	0.08
*Arctonyx collaris* F. Cuvier in É. Geoffroy Saint-Hilaire & F. Cuvier, 1825	Hog Badger	VU	-	377	4.73
** Viverridae **					
*Paguma larvata* (Griffith, 1822)	Masked Palm Civet	LC		515	6.47
** Herpestidae **					
*Urva urva* (B.H. Hodgson, 1836)	Crab-eating Mongoose	LC		19	0.24
** ARTIODACTYLA **					
** Suidae **					
*Sus scrofa* Linnaeus, 1758	Wild Boar	LC	-	1,420	17.83
** Cervidae **					
*Muntiacus reevesi* (W. Ogilby, 1839)	Reeves's Muntjac	LC	-	3,480	43.70
*Muntiacus crinifrons* (P.L. Sclater, 1885)	Black Muntjac	VU	I	318	3.99
** Bovidae **					
*Capricornis sumatraensis* (Bechstein, 1799)	Serow	VU	II	113	1.42
** PRIMATES **					
** Cercopithecidae **					
*Macaca mulatta* (E.A.W. von Zimmermann, 1780)	Rhesus Macaque	LC	II	5	0.06
*Macaca thibetana huangshanensis* Jiang Xuelong & Wang Qishan in Jiang Xuelong, Wang Yingxiang, & Wang Qishan, 1996	Tibetan Macaque	NT	II	797	10.01
** RODENTIA **					
** Sciuridae **					
*Callosciurus erythraeus* (Pallas, 1779)	Pallas's Squirrel	LC	-	1	0.01
** Hystricidae **					
*Hystrix brachyura* Linnaeus, 1758	Malayan Porcupine	LC	-	497	6.24

**Table 2.** Species α-diversity of mammals in the Jiulongfeng Nature Reserve.

Altitude gradient	Number of cameras	Shannon-Weiner index	Pielou index	Simpson index
Low altitude (400–780 m)	16	1.610	0.594	0.309
Mid-altitude (780–1,210 m)	16	1.687	0.639	0.259
