# Supplementary material for: Camera trap survey of mammal diversity and activity rhythms of threatened species in a subtropical forest of Huangshan Mountain, China
Source: Biodivers Data J. 2026 Feb 19;14:e184017. doi: 10.3897/BDJ.14.e184017 (PMC12946825; doi:10.3897/BDJ.14.e184017)
Supplement: Supplementary material 1 — List of mammal species [file bdj-14-e184017-s001.pdf]

Table S1. List of mammal species recorded at the Jiulongfeng Nature Reserve, Huangshan of Anhui Province.

Species Name	Common Name	Geography		Protect the Category				
		Oriental	Palaearctic	IUCN	CITES	CSRL	National Protected	
**INSECTIVORA**								
**Erinaceidae**								
Erinaceus amurensis	Amur Hedgehog		√	LC	-	-	-	△
**Soricidae**								
Crocidura attenuata	Asian Gray Shrew	√		LC	-	LC	-	☆
**CHIROPTERA**								
**Vespertilionidae**								
Myotis chinensis	Large Mouse-eared Bat	√		LC	-	NT	-	☆
Myotis fimbriatus	Fringed Long-footed Myotis			LC	-	NT	-	○
Myotis altarium	Szechwan Myotis			LC	-	NT	-	○
Myotis pilosus	Rickett's Big-footed Bat			-	-	-	-	○
Eptesicus serotinus	Big Brown Bat			LC	-	LC	-	○
Scotomanes ornatus	Harlequin Bat	√		LC	-	LC	-	○
Pipistrellus abramus	Japanese House Bat	√		LC	-	LC	-	☆
**Rhinolophidae**								
Rhinolophus cornutus	Little Japanese Horseshoe Bat	√		-	-	-	-	○
Rhinolophus pearsoni	Rhinolophus Pearsoni	√		LC	-	LC	-	○
Rhinolophus sinicus	Chinese Horseshoe Bat	√		LC	-	-	-	○
**Hipposideridae**								
Hipposideros pratti	Hipposideros Pratti	√		LC	-	LC	-	○
**PHOLIDOTA**								
**Manidae**								
Manis pentadactyla	Chinese Pangolin	√		CR	II	LC	II	△
**LAGOMORPHA**								
**Leporidae**								
Lepus sinensis	Chinses Hare	√		LC	-	LC	-	☆
**RODENTIA**								
**Sciuridae**								
Callosciurus erythraeus	Red Belly Squirrel	√		LC	-	LC	-	☆
Dremomys pernyi	Perny's Long-nosed Squirrel	√		LC	-	LC	-	○
Tamiops swinhoei	Swinhoe's Striped Squirrel	√		LC	-	LC	-	☆
**Cricetidae**								
Eothenomys melanogaster	Pére David's Vole	√		LC	-	LC	-	○
**Muridae**								
Apodemus agrarius	Striped Field Mouse		√	LC	-	LC	-	☆
Rattus tanezumi	Buffbreasted Rat	√		LC	-	LC	-	☆
Rattus norvegicus	Sewer Rat		√	LC	-	LC	-	☆
Niviventer niviventer	White-bellied Rat	√		LC	-	-		☆
Niviventer fulvescens	Chestnut White-bellied Rat	√		LC	-	LC	-	☆
Rattus nitidus	Himalayan Field Rat	√		LC	-	LC	-	○
Berylmys bowersi	Green Rat	√		LC	-	-	-	○

Leopoldamys edwardsi	Edwards's Giant Rat	√		-	-	LC	-	☆
**Hystricidae**								
Hystrix brachyura	Malayan Porcupine	√		-	-	LC		☆
**CARNIVORA**								
**Canidae**								
Vulpes vulpes	Red Fox		√	LC	III	NT	-	○
Nyctereutes procyonoides	Racoon Dog		√	LC	-	NT		○
Cuon alpinus Lepturus	Kiangsi Dhole		√	EN	II	EN	II	△
**Mustelidae**								
Martes flavigula	Yellow-throated Marten		√	LC	-	-	-	☆
Mustela kathiah	Yellow-bellied Weasel	√		LC	-	NT	-	○
Mustela sibirica	Siberian Weasel		√	LC	III	LC	-	△
Melogale moschata	Chinese Ferret-badger	√		LC	-	NT	-	○
Meles meles	European Badger		√	LC	-	NT	-	☆
Arctonyx collaris	Hog Badger	√		VU	-	NT	-	☆
**Viverridae**								
Viverra zibetha	Large Indian Civet	√		NT	III	VU	II	○
Viverricula indica	Small Indian Civet	√		LC	III	VU	II	○
Paguma larvata	Masked Palm Civet	√		LC	III	NT		☆
Urva urva	Crab-eating Mongoose	√		LC	III	NT		○
**Felidae**								
Prionailurus bengalensis	Leopard Cat	√		LC	II	VU	-	△
Neofelis nebulosa	Clouded Leopard	√		VU	I	CR	I	△
Panthera pardus	Indian Leopard	√		NT	I	EN	I	△
**ARTIODACTYLA**								
**Suidae**								
Sus scrofa	Wild Boar		√	LC	-	LC		☆
**Cervidae**								
Muntiacus reevesi	Reeves's Muntjac	√		LC	-	VU	-	☆
Muntiacus crinifrons	Black Muntjac	√		VU	I	EN	I	☆
Elaphodus cephalophus	Tufted Deer	√		NT	-	VU	-	○
Cervus nippon	Sika Deer	√			-	CR	I	○
**Bovidae**								
Capricornis sumatraensis	Serow	√		VU	I	VU	II	☆
**PRIMATES**								
**Cercopithecidae**								
Macaca mulatta	Rhesus Macaque	√		LC	II	LC	II	△
Macaca thibetana huangshanensis	Tibetan Macaque	√		NT	II	VU	II	☆
**Sum Total of Species**			**n = 52**					

Note: EN: Endangered, VU: Vulnerable, NT: Near Threatened, LC: Least Concern; DD: Data Deficient; ☆: Entities are captured or observed, △: obtained by visiting and investigating, ○: literature review. Source: Data of Huangshan District Forestry Bureau, 2021.
